# A Systematic Review of Interventions to Promote Cervical Cancer Screening among Immigrant Vietnamese Women

**DOI:** 10.1007/s10900-024-01395-w

**Published:** 2024-08-26

**Authors:** Jacqueline Hua, Kristopher Jackson

**Affiliations:** 1 Cancer Prevention Fellowship Program, Division of Cancer Prevention, National Cancer Institute, 9609 Medical Center Dr, Rockville, MD 20850 USA; 2 Center for AIDS Prevention Studies (CAPS), University of California, San Francisco, CA USA; 3 School of Public Health, University of California, Berkeley, CA USA

**Keywords:** Cervical cancer, Screening, Intervention, Vietnamese, Women, Cultural tailoring

## Abstract

Vietnamese women have a higher incidence rate of cervical cancer and are less likely to have ever been screened for cervical cancer than their White counterparts in the US. This review synthesizes findings from published interventions to promote cervical cancer screening in this vulnerable population. Articles were identified through a systematic search of PsycInfo, Embase, Pubmed, Web of Science, and the Cochrane Register of Controlled Trials in October 2022. Articles were included if they were published in a peer-reviewed journal, written in English, included one or more interventions promoting cervical cancer screening, assessed at least one outcome relevant to screening, and included a sample of *≥* 70% Vietnamese participants. Quality assessment scores were computed using the Downs and Black Checklist. Fifteen articles met review inclusion criteria. Studies were, on average, of good quality. Most studies were conducted in the US (*n* = 12), used a quasi-experimental design (*n* = 9), and employed multiple intervention strategies (*n* = 12). Intervention strategies included educational sessions, lay health worker (LHW) outreach, small media, mass media, patient navigation, and community or healthcare-based strategies. The most common study outcomes were screening intention and receipt. All but two studies reported improved cervical cancer screening outcomes following intervention. Findings support the effectiveness of multicomponent culturally tailored interventions to improve cervical cancer screening outcomes in immigrant Vietnamese women. Further research is needed to determine whether these interventions will be as successful in non-US countries and to address broader community- and healthcare-based factors in screening.

## Introduction

Cervical cancer is the fourth leading cause of cancer deaths among women globally [1]. In 2018, approximately 311,000 women died from cervical cancer worldwide [2]. Cervical cancer screening is known to reduce mortality [3, 4]. The American Cancer Society recommends women ages 25 years old and older undergo screening every three years [5]. However, in 2019, only 76.4% of screening-eligible women in the US had been screened for cervical cancer in the last three years [6]. Aside from the US, only 33% of women in low- and middle-income countries reported ever being screened for cervical cancer in their lifetime [7].

One group that is disproportionately at risk for cervical cancer is Vietnamese women [8]. Despite a lower cervical cancer incidence among Asian women overall as compared to non-Hispanic White women, the incidence of cervical cancer among Vietnamese American women is 9.5 per 100,000 versus 6.8 per 100,000 White women in the US [8]. Cervical cancer incidence is also higher for Vietnamese women than it is for Pakistani (4.2 per 100,000), Chinese (4.5 per 100,000), Japanese (5.8 per 100,000), Filipino (7.0 per 100,000), Hawaiian (6.7 per 100,000), or Korean (7.5 per 100,000) women in the US [8]. Further, research suggests only 53% of Vietnamese American women have ever been screened for cervical cancer as compared to 85% of non-Hispanic White women in the US [9]. In Vietnam, screening rates are even lower, with an estimated 25% of women reporting having ever been screened [10].

Numerous psychosocial factors contribute to low cervical cancer screening rates among Vietnamese women such as inadequate knowledge about screening, anxiety about receiving an abnormal test result, concerns with modesty, and preference for traditional Asian medicine over Western medicine [9, 11–13]. Structural factors such as difficulty with navigating the US healthcare system are also barriers to cervical cancer screening for immigrant Vietnamese women [13]. For instance, not being able to identify a female provider is associated with lowered intentions to screen and limited English proficiency is associated with lowered awareness of screening among Vietnamese women [14, 15]. Similarly, many Vietnamese women cite structural factors such as lack of insurance and having to travel too far as a reason for not screening [13].

Despite the increased incidence of cervical cancer among Vietnamese women and the well-established barriers to cervical cancer screening in this population, there is no consensus regarding the best-practices for increasing cervical cancer screening in this population. Lu et al. [16] conducted a review of interventions to promote breast and cervical cancer screening among Asian women, but the authors suggested that intervention effectiveness varies by Asian subgroup. Further, the authors did not describe the interventions best-suited for Vietnamese women. Another review of targeted screening interventions for minoritized racial/ethnic women conducted by Glick et al. [17] concluded patient navigation was successful in increasing cervical cancer screening. Findings from a meta-analysis by Han et al. [18] support multi-intervention approaches for increasing screening among minoritized racial/ethnic women. However, neither work makes specific recommendations for Vietnamese women. Staley et al. [19] reviewed interventions promoting cervical cancer screening and study findings supported the use of invitation letters. Though comprehensive, this review did not focus on Vietnamese women.

Aggregating data on cervical cancer screening outcomes of Asian subgroups masks important group disparities [20]. Moreover, screening disparities between Asian subgroups stem from heterogeneity in perceived screening barriers [21, 22]. Past work has identified differences in associations between language barriers and cancer screening rates among Chinese, Korean, Cambodian, and Vietnamese women [21]. Reviewing interventions that target any minoritized racial/ethnic groups can also be problematic given each group may face different barriers to screening. For example, Asian and Latina women differ in average income and education—both of which are known to affect screening rates [23]. Thus, conclusions from past reviews of interventions targeting Asian or other minoritized racial/ethnic women may not apply to Vietnamese women and a systematic review is needed to inform best approaches for this group. To bridge this gap in the literature, this review provides a synthesis of interventions to promote cervical cancer screening among immigrant Vietnamese women.

## Methods

### Search Strategy

This review followed PRISMA guidelines and was conducted with the assistance of Covidence software [24]. As this review synthesized data from existing published works, it was exempt from ethical review. PsycInfo, Embase, Pubmed, Web of Science, and the Cochrane library were queried in October 2022. Search terms included: cervical cancer screening AND intervention AND Vietnamese, cervical cancer test AND intervention AND Vietnamese, pap test AND intervention AND Vietnamese, pap smear AND intervention AND Vietnamese, HPV test AND intervention AND Vietnamese, human papillomavirus test AND intervention AND Vietnamese. A backward reference search of the meta-analysis and review articles cited here [16–19] was also conducted.

### Study Selection and Data Extraction

**Fig. 1:**
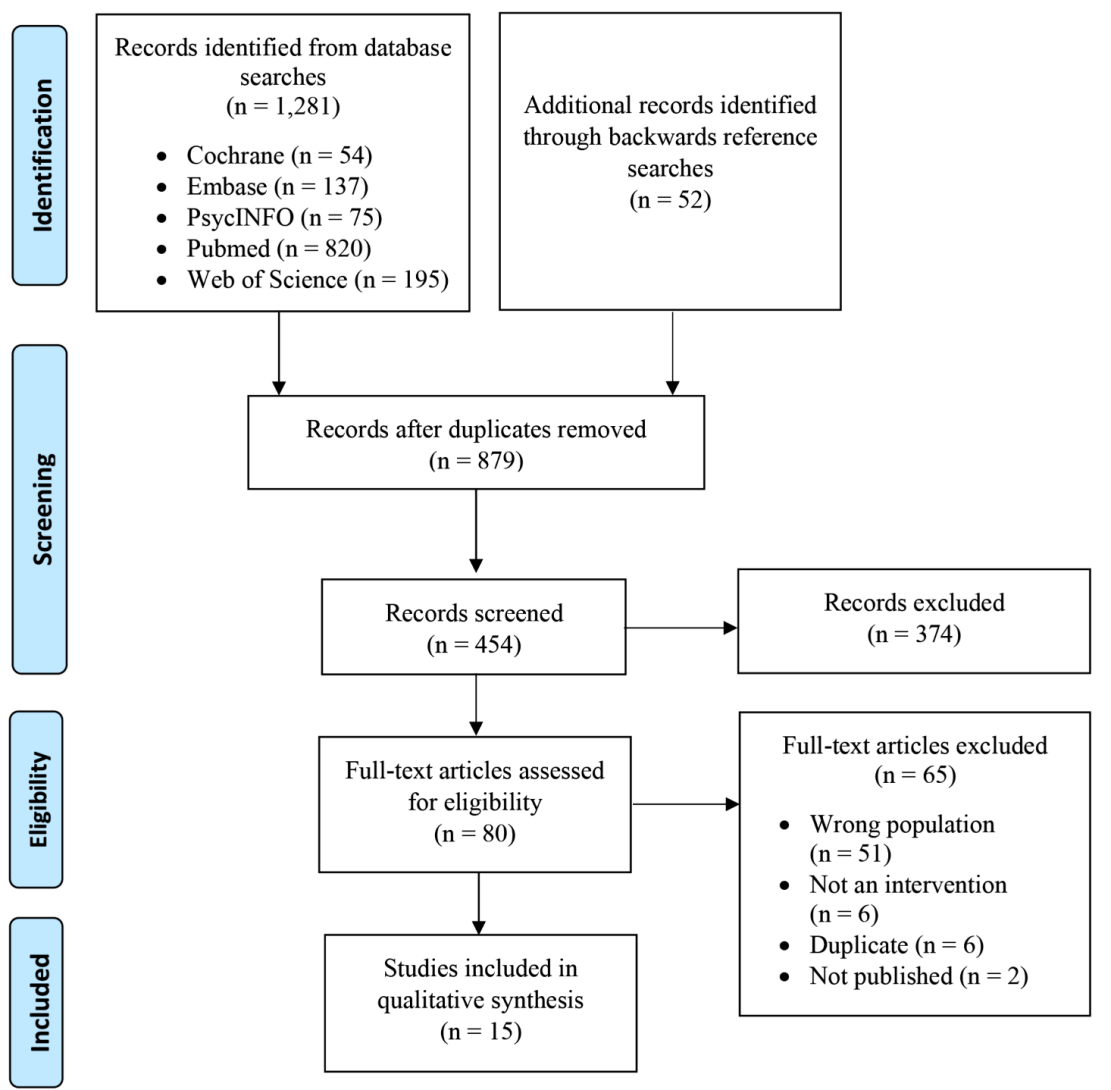
PRISMA flow diagram displaying article identification and screening procedures

Figure 1 presents study identification and screening procedures. Studies were included if they met the following criteria: (1) published in a peer-reviewed journal, (2) written in English, (3) included at least one intervention to improve cervical cancer screening outcomes, (4) assessed at least one outcome relevant to cervical cancer screening (e.g., intentions to screen, screening behavior), and (5) included a sample of *≥* 70% Vietnamese participants. The search yielded 1,333 articles. After removing 879 duplicates entries, 454 articles were considered for inclusion.

Titles and abstracts were screened by one author. After title and abstract screening, 374 articles were excluded. Two authors then independently conducted full-text screening of the remaining 80 articles and consensus was achieved through discussion. After full-text screening, 65 articles were excluded, yielding a final sample of 15 articles. The following information was extracted: first author, year, study design, setting, sample characteristics, sample size, intervention strategies, screening outcomes assessed, and findings relevant to screening outcomes. Due to the heterogeneity in the intervention strategies and outcome measures, it was not appropriate to perform a meta-analysis and study data were qualitatively synthesized.

### Quality Assessment

To assess the quality of each study, we used a modified version of the Downs and Black checklist [25]. The checklist includes items for reporting, external validity, internal validity, and power. Reporting assesses whether authors provided sufficient information to allow readers to make unbiased assessments of study findings. External validity assesses the extent to which findings can be generalized to the population from which participants were derived. Internal validity consists of two subcategories—bias and confounding—which assess measurement bias and selection bias. As in previous research [26], we modified the item for power to assess whether a power analysis was reported (scored as 1 = yes, 0 = no). Using the modified checklist, studies could achieve a maximum score of 28. Consistent with past reviews [27], quality was scored as follows: very good (*≥* 20), good (15–19), fair (11–14), and poor (*≤* 10). Scores for each study were independently computed by two authors and consensus was achieved through discussion.

## Results

### Overview of Studies

Overall, 15 studies were included in the final sample. Table 1 presents the samples, study designs, and intervention strategies used in all studies. Twelve studies were conducted in the US [28–39], as well as one in Australia [40], one in Taiwan [41], and one in Korea [42]. Participants were mostly comprised of immigrant Vietnamese women in the US with ages ranging from 18 to 102. Sample size ranged from 21 to 3,575 participants, with a total of 9,067 participants across all studies. Among studies that were conducted in the US, the majority took place in California. Nine studies used a quasi-experimental or pre-post design [28–31, 34, 37–39, 41], four were randomized controlled trials [32, 33, 36, 40], and two used a pretest-posttest randomized experimental design [35, 42]. Regarding interventions, 11 studies used a combination of multiple strategies while four studies used a single strategy to improve cancer screening outcomes. The most common intervention strategies were educational sessions and lay health worker (LHW) outreach. The most common study outcomes were intention to screen and screening receipt. Only two studies [35, 40] did not report improvements to any cervical cancer screening outcomes following intervention.

**Table 1:** Sample and design of studies to improve cervical cancer screening outcomes in Vietnamese women

First Author (Year)	Target Population		Sample Size	Study Design	Setting	Intervention Strategies
Bird et al. (1998)	Immigrant Vietnamese Women		Total *N* = 717 *n* = 345 intervention *n* = 372 control	Quasi-Experimental	San Francisco (intervention) and Sacramento (control), California, US	• Mass Media • Education • Lay health Workers
Del Mar et al. (1998)	Immigrant Vietnamese Women		Total *N* = 689 *n* = 359 intervention *n* = 330 control	Randomized Controlled Trial	Brisbane, Australia	• Mass Media • Small Media
Jenkins et al. (1999)	Immigrant Vietnamese Women		Total *N* = 1,809 *n* = 905 intervention *n* = 904 control	Quasi-Experimental	Alameda/Santa Clara Counties (intervention), and Los Angeles/Orange Counties (control), California, US	• Mass Media
Lam et al. (2003)	Immigrant Vietnamese Women		Total *N* = 400 *n* = 200 intervention *n* = 200 control	Quasi-Experimental	Santa Clara County, California, US	• Mass Media • Education • Lay Health Workers
Nguyen et al. (2006)	Immigrant Vietnamese Women		Total *N* = 3,575 *n* = 1,566 intervent. *n* = 2,009 control	Quasi-Experimental	Santa Clara County, California (intervention) and Harrison County, Texas (control), US	• Mass Media • Small Media • Education • Lay Health Workers • Patient Navigation • Community and Healthcare-Based Strategies
Mock et al. (2007)	Immigrant Vietnamese Women		Total *N* = 1,005 *n* = 491 intervention *n* = 477 control	Randomized Controlled Trial	Santa Clara County, California, US	• Mass Media • Education • Lay Health Workers • Patient Navigation
Taylor et al. (2010)	Immigrant Vietnamese Women		Total *N* = 174 *n* = 84 intervention *n* = 90 control	Randomized Controlled Trial	Seattle, Washington, US	• Small Media • Education • Lay Health Workers
Nguyen et al. (2011)	Immigrant Vietnamese Women		*N* = 21	Quasi-Experimental	Southeastern city in the US	• Small Media • Education • Lay Health Workers
Nguyen et al. (2014)	Immigrant Vietnamese Women		Total *N* = 102 *n* = 51 intervention *n* = 51 control	Pre-test Post-test Experiment	Richmond, Virginia, US	• Small Media • Education • Lay Health Workers
Ma et al. (2015)	Immigrant Vietnamese Women		Total *N* = 1,416 *n* = 758 intervention *n* = 658 control	Randomized Controlled Trial	30 US communities	• Small Media • Education • Patient Navigation
Lee et al. (2017)	Married Immigrant Vietnamese women		Total *N* = 260 *n* = 130 intervention *n* = 130 control	Quasi-Experimental	Southern Taiwan	• Small Media • Lay Health Workers
Fernandez-Esquer et al. (2020)	Vietnamese Nail Salon Workers		*N* = 186	Quasi-Experimental	Houston, Texas, US	• Small Media • Education • Lay Health Workers • Patient Navigation
Kim et al. (2020)	Married Immigrant Vietnamese Women		Total *N* = 58 *n* = 24 experimental *n* = 24 control	Pre-test Post-test Experiment	South Korea	• Education
Duong et al. (2021)	Vietnamese Family Members		*N* = 41	Quasi-Experimental	Orange County, California, US	• Lay Health Workers
Fricovsky et al. (2022)	Immigrant Vietnamese Women		*N* = 120	Quasi-Experimental	San Diego, California, US	• Education

### Study Quality

Study quality scores ranged from 11 to 20 (*M* = 16.67; Table 2), suggesting studies to promote cervical cancer screening outcomes in Vietnamese women are, on average, of good quality. Studies scored the highest, on average, in the reporting category, indicating adequate reporting of study objectives, methodology, and results. Studies also fared well regarding measurement bias. Average scores in the bias subcategory for internal validity indicate low presence of measurement bias. Conversely, studies scored the lowest, on average, in power. Only five studies in the sample (33%) reported a power analysis, suggesting the need for more transparency regarding sample size planning in this area of research. Studies also exhibited a low average score for external validity indicating limited generalizability of findings. The average score in the confounding subcategory for internal validity suggested mixed findings regarding the presence of bias in participant selection. This is perhaps unsurprising given most studies were conducted in the US and multiple studies included specific subsamples of Vietnamese women (e.g., women recruited from churches).

**Table 2:** Quality Assessment of Studies using the Downs and Black Checklist

First Author (Year)	Reporting Score (out of 11)	External Validity Score (out of 3)	Internal Validity – Bias Score (out of 7)	Internal Validity – Confounding Score (out of 6)	Power (out of 1)	Total Score (out of 28)
Bird et al. (1998)	8	2	4	3	1	18
Del Mar et al. (1998)	5	2	5	3	0	15
Jenkins et al. (1999)	9	2	4	4	1	20
Lam et al. (2003)	9	0	4	2	0	15
Nguyen et al. (2006)	9	2	4	4	1	20
Mock et al. (2007)	10	0	4	4	0	18
Taylor et al. (2010)	9	1	5	3	0	18
Nguyen et al. (2011)	6	0	4	1	0	11
Nguyen et al. (2014)	9	1	5	5	0	20
Ma et al. (2015)	10	1	4	4	0	19
Lee et al. (2017)	9	1	4	2	1	17
Fernandez-Esquer et al. (2020)	7	2	3	3	0	15
Kim et al. (2020)	6	0	5	2	1	14
Duong et al. (2021)	7	1	4	2	0	14
Fricovsky et al. (2022)	8	1	4	3	0	16
**Overall Mean**	8.07	1.07	4.20	3.00	0.33	16.67

### Intervention Strategies

Table 3 presents intervention descriptions, outcomes assessed, and main findings from each study.

**Table 3:** Description of interventions, outcomes, and main findings

First Author (Year)	Description of Intervention	Outcomes	Main Findings
Bird et al. (1998)	• Lay health worker educational sessions • Educational materials • Promotional events	• Awareness of screening • Screening receipt • Screening maintenance	Screening recognition, receipt (i.e., ever been screened), and maintenance (i.e., having received two screening tests within the previous 5 years) significantly increased from pre- to posttest in the intervention community. Recognition of screening decreased and there were no differences in screening receipt nor maintenance from pre- to posttest in the control community.
Del Mar et al. (1998)	• Screening invitation letters • Mass media campaign	• Screening receipt 1 year post-intervention	There were no differences in screening receipt between participants in the intervention and control groups.
Jenkins et al. (1999)	• Mass media campaign	• Screening recognition • Intention to screen • Screening receipt • Screening currency	Recognition of screening in the intervention area significantly increased from pre- to posttest (62–69.5%). However, intention (i.e., planning) to screen significantly declined from pre- to posttest. Screening receipt (i.e., ever having been screened) and screening currency (i.e., being up to date with screening) remained unchanged. Those who lived in the intervention area at posttest had significantly greater odds of recognizing screening and intention to screen.
Lam et al. (2003)	• Lay health worker educational sessions • Mass media campaign	• Awareness of screening • Intention to screen • Screening receipt between pre- and post-intervention	Post-intervention, more women in the combined lay health worker outreach and media campaign group exhibited increased awareness of screening as compared to women in the media campaign only group. Screening intention and receipt significantly increased in the combined intervention group, but not the media only group.
Nguyen et al. (2006)	• Mass media campaign • Lay health worker educational sessions • Patient navigation • Registry and reminder system • Continuing education for Vietnamese physicians • Restoration of a cervical cancer control program	• Awareness of screening • Ever been screened • Recency of screening • Intention to screen	In the intervention community, there were significant increases in awareness of screening, screening receipt (i.e., ever been screened), recency of screening (i.e., screening within 12 months), and intention to screen among women who had never been screened from pre- to posttest. In the comparison community, awareness decreased from pre- to posttest and changes in receipt and recency were not significant. Intention to screen significantly decreased in the comparison community from pre- to posttest.
Mock et al. (2007)	• Lay health worker educational sessions • Patient navigation • Mass media campaign	• Ever been screened • Up to date with screening • Awareness of cervical cancer • Knowledge of who should obtain screening • Intention to screen	Screening receipt (i.e., ever been screened), up to date with screening (i.e., obtained a first screening or obtained screening after a lapse of more than 1 year), awareness of cervical cancer, and knowledge of screening all increased among participants in the combined intervention and media-only group, but significantly more so in the combined group. Increases in intention to screen among women who had never been screened did not differ between the groups.
Taylor et al. (2010)	• Lay health worker educational sessions • Educational materials	• Screening receipt within 6 months post-intervention	Ever-screened women in the intervention group were more likely to report screening than were ever-screened women in the control group. There were no differences in screening receipt between women who had never been screened in the intervention and control groups.
Nguyen et al. (2011)	• Lay health worker educational sessions • Take-home reading materials • Referral to local providers	• Screening self-efficacy • Intention to screen • Screening receipt	From pre- to posttest, participants reported significant increases in self-efficacy for screening. Additionally, screening intention increased from 71–100% and 19% of participants who were not up to date with screening reported receiving screening during the study period.
Nguyen et al. (2014)	• Lay health worker educational sessions • Take-home materials • Referral to local providers	• Attitudes toward screening • Screening receipt within 6 months post-intervention	There were no differences in attitudes toward screening nor 6-month screening receipt between the intervention and control groups.
Ma et al. (2015)	• Educational sessions • Patient navigation • Physician-patient communication training • Referral to screening sites • Screening reminders	• Screening receipt within 12 months post-intervention • Intention to screen	There was a significant difference in rate of screening within the 12-month follow-up period between the intervention (60.1%) and control (1.6%) groups. Significantly more women who did not screen during the follow-up period reported intentions to screen in the intervention group than the control group.
Lee et al. (2017)	• Health education brochure • Telephone consultation as needed	• Knowledge of screening • Barriers to screening • Intention to screen	At 3 and 6 months postintervention, the intervention group was more likely to have greater knowledge of screening, lower perceived barriers to screening, and greater intention (i.e., willingness) to screen than the control group.
Fernandez-Esquer et al. (2020)	• Lay health worker educational sessions • Cancer screening brochures • Patient navigation	• Adherence to screening guidelines (screening within last 3 years)	Non-adherent participants who accepted navigation services were significantly more likely to report screening at follow-up (84%) as compared to participants who did not accept navigation (50%).
Kim et al. (2020)	• Educational sessions	• Benefits of screening • Barriers to screening • Intention to screen	Participants in the intervention group reported significantly lower perceived barriers to screening and greater intentions to screen as compared to those in the control group.
Duong et al. (2021)	• Family group chat led by family health advocates	• Intention to screen	Post-intervention, 50% of female participants aged 21 years or older reported intentions to screen or repeat screening.
Fricovsky et al. (2022)	• Educational sessions	• Knowledge of screening • Planning to screen • Barriers to screening	Participants had improved knowledge about screening from pre- to posttest. There was also a significant increase in participants who planned to screen in the next 12 months from pre- (57%) to posttest (78%). Not knowing where to get screened was the most common barrier to screening.

#### Educational Sessions

Lack of knowledge represents a common screening barrier among Vietnamese women [11]. Consistently, 11 studies in this review included educational sessions as part of their intervention. The educational sessions conducted by Nguyen et al. [34], Nguyen et al. [35], Fernandez-Esquer et al. [37], and Fricovsky et al. [39] were held in community settings. The sessions by Bird et al. [28] and Taylor et al. [33] took place in participants’ homes. The sessions by Nguyen et al. [31] were held in both participants’ homes and community-based organization offices. The locations of the sessions conducted by Lam et al. [30], Mock et al. [32], Ma et al. [36], and Kim et al. [42] were unspecified. All educational sessions were conducted in Vietnamese or had Vietnamese interpreters available for participants, indicating the importance of addressing language barriers. In these sessions, facilitators typically gave presentations on cervical cancer epidemiology, symptoms, risk factors, heightened risk among Vietnamese women, cancer prevention, screening procedures, screening locations, diagnosis, and treatment. Materials presented in educational sessions were tailored to be culturally appropriate. For example, the educational sessions by Kim et al. [42] included stories and illustrations of the incidence of cervical cancer in immigrant Vietnamese women to maximize relatability to their target population of Vietnamese women living in South Korea. All but one study [35] that employed educational sessions reported improved cancer screening outcomes [28, 30–34, 36, 37, 39, 42]. This aligns with prior research suggesting that improving knowledge is critical for promoting cervical cancer screening in Vietnamese women [11]. Nevertheless, these findings should be interpreted with caution as most studies paired educational sessions with other strategies, such as LHW outreach and small media.

#### Lay Health Workers (LHWs)

Some evidence suggests that lay health workers (i.e., community members trained to provide health care services or promote health in their communities) can help deliver health interventions for populations that are hesitant to accept them otherwise [43]. In this review, 10 studies included Vietnamese-speaking LHWs. Eight studies used LHWs to facilitate educational sessions [28, 30–35, 37]. For example, LHWs in the Lam et al. [30] study organized educational sessions about screening in addition to explaining how to access screening services and helping some women schedule appointments. All but one study [35] that incorporated LHWs reported improved cervical cancer screening outcomes [28, 30–33, 35, 37, 38, 41]. This observation aligns with past work demonstrating the effectiveness of LHW interventions in promoting cancer screening among minoritized racial/ethnic groups [44]. Indeed, the inclusion of LHWs in interventions can help address language barriers, provide social support, and build trust between participants and researchers [45–47]. The evidence here supports the use of LHWs in improving cervical cancer screening outcomes among Vietnamese women. With the exception of Duong et al. [38], all studies used LHWs in conjunction with other intervention strategies, such as educational sessions and patient navigation.

#### Small Media

Past work suggests that an effective strategy to increase cancer screening in Asian countries is to distribute small media that promotes screening behavior [48]. In general, small media are aimed at individuals or small groups while mass media are aimed at large numbers of people. In this review, eight studies paired small media with other intervention strategies to improve screening outcomes. For instance, Ma et al. [36] and Nguyen et al. [31] both paired screening reminders with patient navigation services along with other intervention strategies. Small media consisted of educational materials, screening invitations, and screening reminders. Aside from screening reminders, all small media were in Vietnamese. Taylor et al. [33], Nguyen et al. [34], Nguyen et al. [35], and Fernandez-Esquer et al. [37] distributed materials to supplement educational sessions. These typically consisted of culturally-tailored booklets which served to reinforce content from educational sessions as well as provide additional resources. For example, Fernandez-Esquer et al. [37] distributed brochures highlighting culturally-based cancer beliefs, knowledge, and barriers. Lee et al. [41] also distributed brochures with information describing the symptoms and incidence of cervical cancer, screening procedures, groups that are at high risk, screening locations, payment for screening, fatalism, and religious views to explain the effect of fatalism on screening in women. All but one study [40] that distributed small media reported improved cervical cancer screening outcomes [31, 33–37, 41]. Although this is consistent with research which finds that small media are effective in promoting screening in Asian countries [48], these results should be interpreted with caution as all of the studies included here paired small media with other intervention strategies.

#### Mass Media

Six studies used mass media campaigns to promote cancer screening among Vietnamese women. Mass media campaigns included large-scale distribution of printed materials and/or promotional items, messaging via electronic media, and posting of billboards. All mass media campaigns were in Vietnamese. All studies that used mass media campaigns used them in conjunction with other intervention strategies, with the exception of Jenkins et al. [29] who tested the effectiveness of a mass media campaign as a standalone intervention. In the first phase of their media campaign, Jenkins et al. [29] distributed booklets and posters, placed advertisements, printed articles in Vietnamese newspapers, posted billboards, and aired advertisements on Vietnamese television. In the second phase, they distributed brochures, posters, and calendars. Additionally, they printed articles in Vietnamese newspapers, posted billboards, broadcasted videos on television, and broadcasted video advertisements. In both phases, referral lists of low-cost screening services were published in newspapers.

All but one study [40] that used mass media campaigns reported improvements in cervical cancer screening outcomes [28–32]. It is possible that the mass media campaign by Del Mar et al. [40] would have also produced differences in screening if it had been compared to a control group that was not exposed to a media campaign, however, the researchers were unable to make this comparison. Consistent with past work, [49] the mass media campaigns in these studies were culturally tailored to the target population. Campaigns often occurred during Tết—the Vietnamese new year celebration—to promote visibility and were conscious of barriers that Vietnamese women face to screening (e.g., low financial resources and English proficiency). Printed materials and promotional items were also distributed in locations that were frequented by Vietnamese people and messages were broadcasted on Vietnamese television and radio stations. These practices further highlight the importance of incorporating culturally appropriate materials in interventions to promote cancer screening among Vietnamese women.

#### Patient Navigation

Structural barriers such as difficulty navigating healthcare systems contribute to low screening rates in Vietnamese American women [11]. Research suggests patient navigation can help address these barriers, including assisting patients with communicating with providers, scheduling appointments, and securing transportation to appointments [50]. Four studies in this review used patient navigation. Navigation services varied, with one study having LHWs help participants schedule appointments [32] and others having dedicated navigators help participants with multiple aspects of the healthcare system [31, 36, 37]. In all studies, navigation was used in conjunction with other strategies, such as education and small media. Patient navigators assisted participants with translation, appointment scheduling, asking questions during appointments, requesting information about screening, and transportation needs. All studies that incorporated patient navigation produced positive cervical cancer screening outcomes, which suggests that this strategy is effective for screening promotion among Vietnamese women [31, 32, 36, 37]. Notably, two studies that employed multiple strategies specifically highlighted the role of navigation services in the success of their interventions [36, 37] and a body of research supports the efficacy of navigation services in promoting cancer screening [50, 51]. Nevertheless, these results should be interpreted with caution because patient navigation was always paired with other intervention strategies in the studies included in this review.

#### Community and Healthcare-Based Strategies

Beyond individual-level factors, community and healthcare factors also play important roles in cancer screening [52, 53]. However, only one study employed community and healthcare-based strategies to promote screening. Nguyen et al. [31] carried out a multicomponent intervention in which they hosted educational seminars on cervical cancer for Vietnamese physicians in Santa Clara County, California. These seminars produced significant increases in physician knowledge about cervical cancer, screening recommendations, and evaluation of abnormal test results [31]. Additionally, the researchers helped to re-establish a local cancer control program that provided cost-free screenings.

## Discussion

The present review identified 15 interventions to improve cervical cancer screening outcomes among Vietnamese women. Findings support the effectiveness of multicomponent interventions that were culturally tailored. Successful interventions were delivered in Vietnamese or used Vietnamese interpreters, involved Vietnamese community members in their development and implementation, and addressed screening barriers that were specific to the Vietnamese community. Collectively, this aligns with past research which suggests that addressing language barriers and incorporating culturally appropriate educational materials are key components of cultural tailoring in interventions to improve health outcomes in minoritized racial/ethnic populations [54].

Interventions to promote screening through educational sessions, LHW outreach, distribution of small media, mass media campaigns, patient navigation, and community and healthcare-based strategies generally produced favorable screening outcomes among Vietnamese women. This is consistent with past work which concludes that access-enhancing and educational strategies have been successful in improving screening outcomes among Asian and other minoritized racial/ethnic populations [16–18]. However, unlike Staley et al. [19], we did not find evidence to support the use of screening invitation letters for improving screening outcomes. This is perhaps unsurprising given that our search strategy identified studies that were community-specific and may have excluded invitation letter interventions that were implemented in numerous communities simultaneously. Nevertheless, immigrant Vietnamese women face barriers (e.g., low English proficiency, lack of financial resources) that call for strategies beyond invitation to screening [12].

The majority of the studies included in this review used multiple intervention strategies to improve screening outcomes. Educational sessions were paired with lay health workers in eight studies [28, 30–35, 37]. Several studies have successfully used lay health workers as educational session facilitators to improve cancer screening in other minoritized racial/ethnic groups [55]. The findings here suggest that lay health worker-led educational sessions also appear to be effective for promoting cervical cancer screening in Vietnamese women. Another frequent combination of intervention strategies involved educational sessions and small media, which were paired in six studies [31, 33–37]. In these studies, small media typically included supplemental materials for educational sessions, such as books or pamphlets that reinforced topics. Although the use of multiple intervention strategies in the present studies aligns with past work which supports the use of combined interventions to promote screening [16], it was not possible to make direct comparisons between interventions nor was it possible to determine whether any one specific strategy was driving observed effects. Nevertheless, this is the first systematic review to provide a qualitative synthesis of interventions to improve cervical cancer screening outcomes in Vietnamese women and the findings can inform future interventions aimed at this vulnerable population.

### Limitations of Interventions and Future Directions

Published interventions have been largely successful in improving cervical cancer screening outcomes for Vietnamese women, however, there are some important limitations. Most studies were conducted in the US, limiting the generalizability of findings. Vietnamese women who reside in countries with universal healthcare may not experience as many benefits as those in the US from interventions that address cost barriers to screening. Moreover, screening rates tend to be lower in developing countries [56], highlighting the need for these intervention strategies to be trialed in these settings. In particular, interventions to promote cervical cancer screening are needed in Vietnam, where only 25% of women reporting having ever been screened in their lifetime [10]. Future research can aim to determine whether low screening rates among Vietnamese women who emigrate to the US are attributable to lack of screening availability, barriers to screening, or negative experiences with screening in Vietnam.

Issues with outcome definitions also presented limitations. Interventions often targeted the same outcomes but defined those outcomes differently. Although both Lee et al. [41] and Jenkins et al. [29] measured screening intentions, Lee et al. [41] defined intention as willingness to screen while Jenkins et al. [29] defined intention as planning to get screened. Similarly, Bird et al. [28] and Ma et al. [36] both measured screening receipt. Yet, Bird et al. [28] defined receipt as having ever been screened while Ma et al. [36] defined it as obtaining screening within 12 months of the intervention. Conversely, similar constructs were labeled differently across studies. For example, Mock et al. [32] measured awareness of screening and Jenkins et al. [29] measured recognition of screening. Inconsistencies in how outcomes were defined likely influenced findings and there is a need for standardization of outcome definitions across interventions.

Although results suggest mass media is an effective strategy for promoting screening, it remains unclear whether media campaigns are effective as standalone interventions. Specifically, two studies which compared the effect of media campaigns alone and media campaigns with LHW outreach found that combined interventions produced more favorable outcomes [30, 32]. Yet, another study did not find any differences between a combined mass media and screening letter intervention versus a mass media alone intervention [40].

Despite research implicating the roles of community and healthcare-based factors in cancer screening [52, 53], only one study in this review addressed these factors [31]. Future interventions should work to address other community and healthcare-based factors that may also influence screening uptake such as promoting positive community norms surrounding cervical cancer screening or providing cultural competency training for physicians in hospitals that serve primarily Vietnamese patients.

### Limitations of the Present Review

Although the findings from this review make important contributions to the literature on cervical cancer screening among Vietnamese women, we note some limitations of our approach. First, although we conducted a comprehensive search, there were a limited number of articles that were eligible for inclusion in our sample. It is possible that additional articles would have been included but did not meet inclusion criteria due to lack of specificity regarding the race/ethnicity of their samples. In particular, studies that took place in non-US countries often did not provide information about the racial/ethnic breakdown of their samples and, thus, we were unable to determine whether they recruited Vietnamese participants. Additionally, we did not exclude studies based on specific screening outcomes or study designs, making it difficult to make direct comparisons between interventions. We opted not to exclude studies given the limited number of cancer screening interventions that target Vietnamese women. This review is intended to describe and assess the intervention strategies that have been employed to promote screening in Vietnamese women as well as identify gaps that still need to be addressed. Future reviews can consider synthesizing data from interventions with specific designs or outcomes as more interventions are published in the literature.

## Conclusion

Vietnamese women have a disproportionately higher risk of cervical cancer and lower screening rates than many other racial/ethnic groups in the US. The present review synthesized data from 15 studies to improve cervical cancer screening outcomes among Vietnamese women. Results demonstrate the importance of culturally tailored approaches and the effectiveness of educational sessions about cancer and screening, outreach via LHWs, distribution of small media, mass media campaigns, patient navigation services, and community and healthcare-based intervention strategies in improving screening outcomes for this population. Further research is necessary to determine whether these intervention strategies are successful in non-US settings, to examine whether mass media campaigns are sufficient to promote screening, and to address other community and healthcare factors related to screening.
